# A tandem reinforcement learning framework for localized prostate cancer treatment planning and machine parameter optimization

**DOI:** 10.1002/mp.70306

**Published:** 2026-01-30

**Authors:** Nathan Shaffer, Avinash Reddy Mudireddy, Joel St‐Aubin

**Affiliations:** ^1^ Department of Biomedical Engineering University of Iowa Iowa City Iowa USA; ^2^ The Iowa Initiative for Artificial Intelligence University of Iowa Iowa City Iowa USA; ^3^ Department of Radiation Oncology University of Iowa Iowa City Iowa USA

**Keywords:** artificial intelligence, automatic treatment planning, machine parameter optimization, reinforcement learning

## Abstract

**Background:**

Volumetric modulated arc therapy (VMAT) machine parameter optimization (MPO) is a complex, high‐dimensional problem typically solved with inverse planning solutions that are both temporally and computationally expensive. While machine learning techniques have been explored to automate this process, they often supplement rather than replace conventional optimizers and are fundamentally limited by the quality and diversity of training data. Reinforcement learning (RL) offers a promising alternative, finding optimal strategies through trial‐and‐error by maximizing a narrowly tailored reward function, which can potentially discover novel solutions beyond mimicking features present in existing plans.

**Purpose:**

The purpose of this study was to develop and validate a deep reinforcement learning‐based VMAT MPO algorithm capable of automatically generating clinically comparable treatment plans for prostate cancer that meet machine constraints, entirely independent of a commercial treatment planning system (TPS) optimizer.

**Methods:**

A dataset comprised of 100 prostate cancer patients planned using the criteria from PACE‐B SBRT arm serve as the basis for network training using a 70‐10‐20 training/validation/testing split. An RL framework using a Proximal Policy Optimization (PPO) algorithm was developed to train two tandem convolutional neural networks that sequentially optimize multi‐leaf collimator (MLC) positions and monitor units (MUs) using current dose, contoured structure masks, and current machine parameters as inputs. Training was designed to predict MLC positions and MUs that maximize a dose‐volume histogram (DVH)‐based reward function tailored to prioritize meeting clinical objectives. The fully trained networks were executed on a test set of 20 patients and compared to reference plans optimized with a commercial TPS.

**Results:**

The RL algorithm generated plans in an average of 6.3 ± 4.7 s. Compared to the reference plans, the RL‐generated plans demonstrated improved sparing for both the bladder and rectum across their respective dosimetric endpoints. When normalizing to 95% coverage, the RL generated plans resulted in a statistically significant increase in the PTV $D_{2\%}$, while achieving a significantly reduced $D_{mean}$ for the rectum. All RL plans successfully satisfied all clinical objectives used to optimize the reference plans.

**Conclusions:**

We successfully developed and validated a deep RL framework for VMAT MPO. The algorithm rapidly generates VMAT prostate cancer treatment plans that meet clinical constraints and are dosimetrically comparable to manually optimized plans without the use of a commercial TPS optimizer. This work demonstrates the feasibility of RL as a tool to fully automate the VMAT planning process, offering the potential to decrease planning times while maintaining plan quality.

## 1 INTRODUCTION

Volumetric Modulated Arc Therapy (VMAT) is an advanced form of radiation therapy that delivers highly conformal dose distributions by continuously modulating the beam as the gantry rotates around the patient.^1^ A critical aspect of VMAT delivery is machine parameter optimization (MPO), which involves defining a sequence of machine parameters, specifically the multi‐leaf collimator (MLC) positions and monitor units (MUs), at each control point (CP) over the range of an arc. MPO is a high‐dimensional, constrained problem and is usually handled with a variety of inverse planning solutions.^2^ These methods typically involve minimizing a cost function defined by chosen dose‐volume objectives and weights. Although GPU‐acceleration has greatly increased the efficiency of these methods,^3^, ^4^, ^5^ inverse planning remains highly sensitive to the choice of dose objectives and their relative weights necessitating iterative, expert‐driven weight tuning. This trial‐and‐error workflow contributes to prolonged planning times and inter‐planner variability. The limitations are especially acute in adaptive radiotherapy and real‐time planning, where plans must be generated or re‐optimized rapidly in response to daily anatomical changes. Consequently, there is a clear need for planning methods that are accurate, clinically robust, and fast enough for seamless integration into modern treatment workflows.^6^, ^7^

To overcome these challenges, there have been many attempts to automate the VMAT planning process such as automated rule implementation, knowledge‐based planning, and multicriteria optimization (MCO).^8^ Within these categories, Artificial Intelligence (AI)‐based supervised learning algorithms have been used to predict dose distributions^9^, ^10^, ^11^, ^12^ and objective function weights.^13^, ^14^ While improving the overall planning process, these methods typically only simplify the dose‐objective and weight defining steps, meaning that they still rely on the same VMAT MPO algorithms used in manual planning to generate a deliverable plan. Additionally, supervised learning methods are fundamentally constrained by the quantity, quality, and diversity of the training data. Because they are designed to reproduce features present in the training plans, these models are bound on achievable plan quality and may struggle to generalize to novel patient anatomies or changes in clinical goals.^15^

In contrast to supervised learning methods that aim to mimic existing examples, reinforcement learning (RL) learns by maximizing a reward indicative of desirable outcomes. An agent interacts with an environment by taking actions, receives feedback in the form of a reward, and learns over time which actions maximize rewards.^16^ Through this trial‐and‐error learning process, the agent incrementally improves the strategies it uses to best satisfy its specific goals in a wide range of domains.^17^, ^18^, ^19^ These strategies are generally referred to as the policy, or mapping from states to actions, designed to maximize the cumulative reward over time. Instead of acting as a one‐off optimizer, a generalized policy can utilize a reward that is tailored to represent the desired clinical dose goals, suggesting potential success in treatment planning workflows over a wider range of scenarios.

RL has shown promise for automating radiotherapy planning by adjusting tunable treatment planning system (TPS) parameters in the optimization process such as penalty weights for target coverage and organ‐at‐risk (OAR) sparing as well as dose thresholds.^20^ In this “virtual planner” setting, the agent interacts with a TPS, observes dose‐volume histograms (DVHs) or other plan‐quality metrics, and updates parameters to improve a reward tied to clinical objectives. This strategy has yielded strong results in intensity modulated radiation therapy (IMRT) for localized prostate cancer,^21^, ^22^ stereotactic body radiotherapy (SBRT),^23^ and high‐dose‐rate (HDR) brachytherapy,^24^ but to our knowledge only non‐RL approaches have been used for VMAT.^25^

RL has also been used in HDR brachytherapy where RL agents directly predict source dwell times and positions to shape dose distributions.^26^ In a similar fashion, RL has seen some success for direct VMAT MPO where the agent iteratively configures MLC leaf positions and beam intensities (MUs) across CPs to maximize a dosimetry‐based reward. In this work presented by Hrinivich et al. for VMAT MPO, the agent was trained to output deliverable parameter sets but needed to invoke a TPS in the final stage to achieve clinically comparable plans.^27^ In this work, we develop and validate an RL‐based VMAT MPO algorithm that automatically generates clinically comparable treatment plans that meet machine constraints without a commercial TPS optimizer.

## 2 METHODS

### 2.1 VMAT for MR‐Linac

We developed and evaluated our RL‐based VMAT MPO network to predict machine parameters to be delivered using the Elekta Unity MR Linac using a VMAT delivery model in a research build of the Monaco TPS (Elekta AB, Stockholm, Sweden). While VMAT is not yet clinically available on the Unity, it is shown to be technically feasible.^28^ The dataset comprised 100 prostate cancer patients, with a training‐validation‐testing split of 70‐10‐20. Retrospective patient data used in this study was reviewed and approved by the University of Iowa IRB (IRB00000099) under application 201805779. The set of reference VMAT plans were generated in Monaco based on the SBRT arm of the PACE‐B clinical trial^29^ prescribing 36.25 Gy in 5 fractions to the prostate and proximal 1 cm seminal vesicles with a 5 mm PTV margin. The RL‐generated plans used evenly spaced CPs every 2° in a single arc, with a gap between 4–23° to avoid the Unity MR‐Linac cryostat. Since this cohort involves prostate treatments where the target remains mostly centralized in the beam's field of view, only the central 26 leaf pairs were required for field modulation. Given the MLC leaf pair positions and an MU value, dose can be computed with an in‐house algorithm^30^ developed for MR‐Linac use and readily adaptable to non MR‐Linac machines. While initially developed for IMRT dose calculations, this algorithm was adapted for VMAT delivery and used to calculate the dose at each training iteration which is needed to assign rewards to the network and produce the next input state. The dose algorithm was written as a Python package for easy integration with open‐source machine learning packages required for RL network development. The RL algorithm was implemented in Tensorflow 2.12 and trained on a research server with 8 Tesla V100 SXM2 GPUs with 32 GB of memory each, and an Intel Xeon Platinum 8268 Processor 24‐core CPU with 384 GB of RAM.

### 2.2 Reinforcement learning

#### 2.2.1 Environment and network definition

The goal of RL can be described as finding a policy that maximizes a cumulative reward in a Markov Decision Process (MDP) that models sequential decision‐making within an environment.^16^ Typically, MDPs are characterized by states ($s_{t}$), actions ($a_{t}$), and resulting rewards ($r_{t}$). Here we denote the policy $\pi_{\theta }$, and approximate it using a neural network with weights denoted by θ. We can then treat VMAT MPO as a finite MDP over an arc discretized into *N* CPs, where the goal is to predict *N* sequential actions that achieve the best quality plan. The network takes the current state $s_{t}$ as an input and provides a predicted action​ $a_{t}$ which is the new set of machine parameters for the next CP. From this predicted action, the new state $s_{t+1}$ can be calculated and a DVH‐based reward $r_{t}$ can be awarded that represents the desired dosimetric objectives. After all CPs are predicted, the final VMAT plan is the set of all N actions predicted by the network. Predicting all N actions for a given batch of patients is one episode.

**FIGURE 1:**
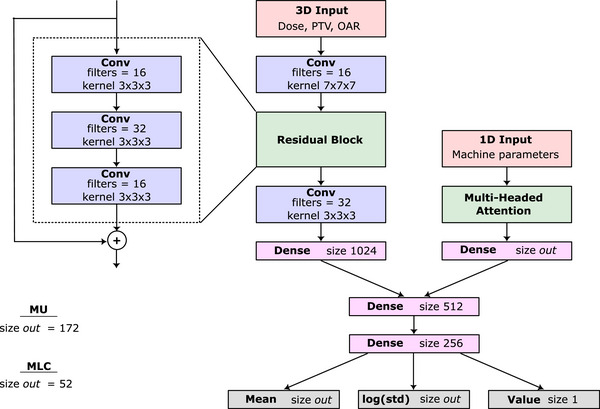
Schematic of the MLC and MU network design. The network accepts a state and returns the action mean, log standard deviation, and value estimate for the input state.

In our case, the policy $\pi_{\theta }$ is represented by two networks working in tandem with the general structure shown in Figure 1. We will denote these as the MLC network $\left(\pi_{\theta_{\mathrm{MLC}}}\right)$ and MU network $\left(\pi_{\theta_{\mathrm{MU}}}\right)$. Both are convolutional neural networks which extract features from the 3D components of $s_{t}$ and combine them with the 1D component where a decoder structure is used to predict (1) the action means, (2) the log of the standard deviation of the action, and (3) a value estimate for the input state. Key elements of the network include a residual block of convolutions to extract 3D features and a multiheaded attention block to capture relationships between the machine parameters represented in the 1D input. Each convolutional layer is separated by batch normalization and a ReLu activation function and each dense layer is separated by layer normalization. The MLC and MU networks differ in a few ways. First, input dimensions of both networks are different, detailed more in section 2.3.2. Second, the output shape, denoted size *out* in Figure 1, of the MLC network is of size 52, corresponding to the number of MLC positions predicted per CP (26 top and 26 bottom) positions. The MU network output is of size 172 as it predicts the MU values of all CPs at once. Finally, the MLC network final output layer uses a tanh activation function for the mean output layer since the MLC positions are bounded. The MU network uses a softplus activation to ensure they remain positive. The value and log standard deviation outputs both use a linear activation function.

A tandem network was chosen in this case due to the behavior exhibited by RL training, particularly the exploration steps needed to find an optimal policy. Initially, we used a single network with branching output heads to predict the MLC positions and MU values at each CP. In training, variance is added to each of these outputs to explore the solution space and find more optimal actions, however balancing exploration between the MU and MLC values proved to be incredibly difficult and led to unstable training. The MU network needs to have sufficient exploration to adequately vary the beam intensity between CPs, however large changes in the MU values make the effect of MLC position changes on the reward negligible. For this reason, the learning signal the network received in this framework from the reward was incredibly noisy and resulted in little to no improvement in reward over time. Based on these initial results, we split this problem into two parts to decouple the effects of the MU and MLC on the reward signal and this decoupling led to a significant improvement in training stability.

#### 2.2.2 Rewards and advantages

The reward function was built to summarize the clinical evaluation of a given plan, similar to a typical objective function used in plan optimization. To calculate the reward, the predicted MLC and MU values are used to first calculate the patient dose normalized to 95% target coverage with the prescription dose scaled to 1.0. From this dose, the objective value is calculated using a mean squared error between the calculated dose and the ideal dose value for that structure. The ideal dose was set to 0.0 for OARs and set to 1.0 for the PTV, meaning lower OAR and more uniform PTV dose are always encouraged. This value is then scaled by a factor of f<1 each time the dose for a given structure satisfied an additional constraint detailed in Table 1. The total dose objective value v can then be expressed as

(1) $$v=\sum_{i\varepsilon S}w_{i}{\left(d_{i}-o_{i}\right)}^{2}\cdot {\left(f_{i}\right)}^{n}$$

**TABLE 1:** Constraints, weights, and scaling factors used in the dose objective reward function.

Structure	Weight $\left(w_{i}\right)$	Constraint	Factor (f)
**PTV**	50	$D_{2.0cm^{3}}<135\%$	0.75
**Bladder**	20	$V_{50\%}<40\%$	0.5
**Rectum**	20	$V_{50\%}<50\%$	0.7
		$V_{80\%}<20\%$	

For structure iεS, weights $w_{i}$, dose values $d_{i}$, ideal dose $o_{i}$, and number of constraints satisfied n. The final reward is the difference in objective value between the old and new state, causing larger improvements in dose objective to return higher rewards. When a constraint is met, n increases by 1 causing the dose objective for the respective structure to be reduced by an additional factor of f, resulting in substantial decrease in objective value and increase in reward. These jumps encourage the network to prioritize meeting the chosen constraints since the biggest total reward over an episode comes when the most constraints are met. These constraints were chosen as they directly mimic the highest weighted constraints used to create the reference plans.

While a reward is assigned for each step in the RL process, it is rarely the case that a single step is solely responsible for achieving that reward. Particularly when applied to VMAT arcs, the reward given to the network at a given CP for meeting a dose constraint is dependent on the totality of all previous CPs. Properly assigning reward to individual steps over the entirety of an episode is known as the credit assignment problem, and is commonly addressed using advantages.^16^, ^31^, ^32^ We calculate the advantage using a generalized advantage estimation (GAE)^33^ that uses backward recursion after episode completion to adequately distribute later rewards to earlier steps. At a given step t, the advantage is defined

(2) $$A_{t}^{\left(\lambda \right)}=\sum_{l=0}^{N}{\left(\gamma \lambda \right)}^{l}\;\delta_{t+1}$$

(3) $$\delta_{t}=r_{t}+\gamma \left(1-d_{t}\right)V_{t+1}-V_{t}$$

With reward $r_{t}$, done flag $d_{t}\varepsilon \left\{0,1\right\}$, value estimate from the critic network $V_{t}$ (section 2.2.3), and tunable discount and GAE parameters γ=0.99 and λ=0.95. Using the temporal‐difference residuals $\delta_{t}$, advantages incorporate future rewards and value estimates from the network at each step giving the network a better evaluation of the future consequences of making a chosen action at the current state.

#### 2.2.3 Proximal policy optimization

We trained our networks using a combined actor‐critic proximal policy optimization (PPO) approach.^34^ The policy (actor) network takes in the current state $\;s_{t}$ of the environment and outputs a probability distribution over all possible actions. This distribution is described by two output layers, representing the means and log standard deviations of possible actions. During training, actions $a_{t}$ are sampled from the resulting probability distribution, allowing the network to explore the action space. At inference, the mean action is used directly. The value (critic) network also takes in the current state $\;s_{t}$ and estimates the expected cumulative reward, or value $\;V_{t}$, of being in that state. We combine these networks to share features and differentiate them using unique output layers. As the episode progresses, trajectories are collected that are defined by experiences consisting of corresponding state, action, reward tuples $\left\{s_{t},\;a_{t},\;r_{n}\right\}$ until termination. Once the episode terminates, the advantages $\;A_{t}$ for all steps are computed which are then normalized by batch and used in the PPO loss shown in equation 4.

(4) $$L_{t}^{PPO}\;\left(\theta \right)={\hat{E}}_{t}\;\left[L_{t}^{clip}\left(\theta \right)-c_{1}L_{t}^{VF}\left(\theta \right)+c_{2}S\left[\pi_{\theta }\right]\left(s_{t}\right)\right]$$

The constant values were chosen to be $\;c_{1}=0.45$ and $c_{2}=0.005$. The PPO loss function is designed to be maximized, but in practice, the negative of the loss is minimized.^34^ The PPO loss uses 3 terms to drive network training. The first term, $L_{t}^{clip}\left(\theta \right)$, is a product of the advantage $\;A_{t}$ and the importance ratio $\;R_{t}\left(\theta \right)$, which is the ratio of the probability of taking action $\;a_{t}$ while in state $\;s_{t}$ using the current and old policy weights.

(5) $$L_{t}^{clip}\;\left(\theta \right)={\hat{E}}_{t}\;\left[\min(R_{t}\left(\theta \right)\cdot A_{t},\mathrm{clip}\left(R_{t}\left(\theta \right),\;1-\varepsilon ,\;1+\varepsilon \right)\cdot A_{t}\right],$$

(6) $$R_{t}\left(\theta \right)=\frac{\pi_{\theta }\left(a_{t}|s_{t}\right)}{\pi_{\theta_{old}}\left(a_{t}|s_{t}\right)}\;$$

This value is clipped to avoid large policy updates which can destabilize training. The clipping parameter ε was set to 0.15. The second term in Equation 4, $L_{t}^{VF}\left(\theta \right)$, is the value critic term that measures how accurately the network can predict the value of a state and is simply the mean squared error between the current network predicted value $\;V_{\theta }\left(s_{t}\right)$ and the actual return $\;G_{t}$ which is a function of $A_{t}$ and the old network predicted value $\;V_{\theta_{\mathrm{old}}}\left(s_{t}\right)$.

(7) $$L_{t}^{VF}\;\left(\theta \right)={\hat{E}}_{t}\;\left[{\left(V_{\theta }\left(s_{t}\right)-G_{t}\right)}^{2}\right],$$

(8) $$G_{t}=A_{t}+V_{\theta_{\mathrm{old}}}\left(s_{t}\right)$$

The third term in Equation 4, $S\left[\pi_{\theta }\right]\left(s_{t}\right)$, is an entropy bonus term^35^, ^36^ calculated from the probability distributions predicted by the network defined from the output means and log standard deviations. This term exists to encourage exploration and avoid collapsing into a definite policy prematurely.

In traditional supervised learning, loss functions are designed in a way to satisfy the primary goal of minimizing the distance between the network output and selected target. This is not the case in a PPO loss where instead, we aim to maximize an expected return. For this reason, the PPO loss function was designed to be maximized,^34^ or in practice, the negative of the PPO loss is designed to be minimized to better suit standard optimizers used in network training. Supervised learning also typically compares network outputs to a stationary target, meaning that there is a theoretical lower limit to the loss, usually 0. In contrast, the PPO loss is instead compared to a moving target (Equation 7 and Equation 8) and assumes no limit to the expected return. As a result, there is no theoretical lower limit to the loss. For these reasons, typically the loss is only monitored to assess gradient stability, and the reward is monitored instead to assess the health of network training.

### 2.3 Network training

#### 2.3.1 Initialization

The trial‐and‐error process used in RL training offers unique benefits over other types of machine learning algorithms, however these benefits come at the cost of sample inefficiency and instability inherent to a training system that requires a wide variety of experiences. This problem is exacerbated when dealing with complex, high‐dimensional environments.^16^, ^37^ Imitation learning (IL) has emerged as a way to address these limitations, where supervised learning is used to initialize network weights before the RL training begins.^38^ We used a version of IL called behavioral cloning. Here, the network first learns from the actions of an expert demonstrator to discourage exploring states that are obviously detrimental, leading to faster convergence when RL is subsequently used.^39^, ^40^ A subset of 10 of the MLC positions of the reference VMAT plans were used in the initialization of the MLC network. A mean square‐error loss between these reference MLC positions and the network outputs was minimized over 500 epochs using an Adam optimizer to complete the supervised learning phase of the IL process. The resultant network weights were then used as a starting point for the RL phase, and the MLC positions predicted by the network using these initialized weights were used as the initial states for RL training loop.

#### 2.3.2 RL workflow

The two networks interact with the environment using distinct state and action space definitions tailored to their respective tasks:

- The **MLC Network** operates sequentially per CP. A **state** of this network consists of a (1) three‐channel, three‐dimensional (3D) tensor input (48 × 48 × 128) containing the beam's‐eye‐view (BEV) aligned dose, PTV, and OAR masks, and (2) a 1D vector of length 53 (52 MLC positions + 1 MU). Its **action** is to predict the 52 MLC positions for the subsequent CP.
- The **MU Network** operates globally, for all CPs. A **state** of this network consists of a (1) three‐channel, 3D tensor input (48 × 128 × 128) of cumulative dose and full anatomical masks, and (2) a single comprehensive 1D vector of length 9116 ([52 MLC positions + 1 MU] × 172 CPs). Its **action** is to predict all 172 MU values simultaneously.

**FIGURE 2:**
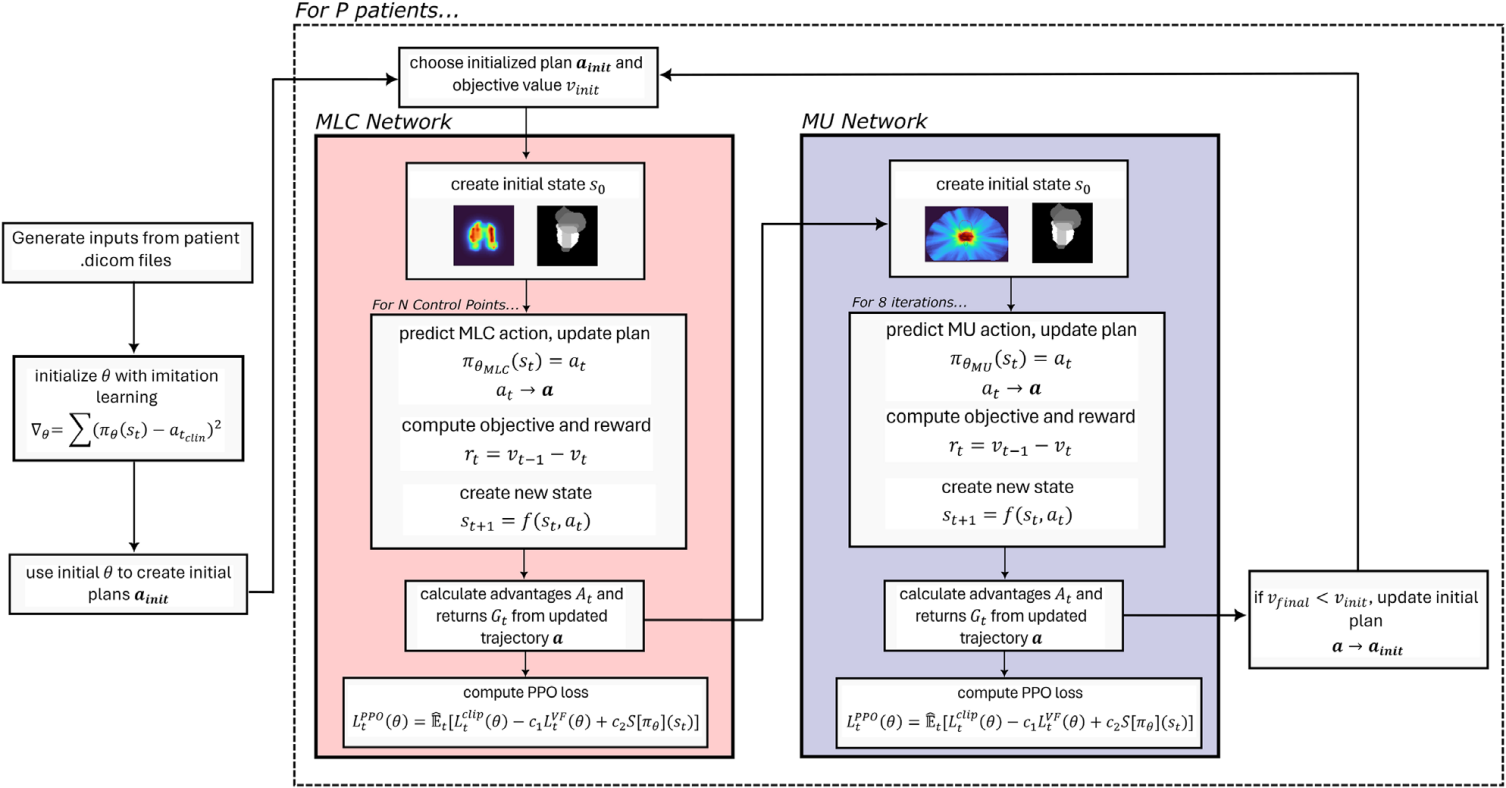
Flowchart demonstrating the entire training process including weight initialization and reinforcement learning updates for both the MLC and MU networks.

Three dimensional inputs were used in both the MLC and MU networks to capture the full dosimetric impacts of the different states predicted by the RL network, and to enable to network to understand the geometric shapes, locations, and proximity of the target and OARs. A flowchart of the major steps of the training algorithm can be seen in Figure 2. MLC training begins first using the IL initialized weights and MLC positions from which an initial state $s_{0}$ is created. The MLC network takes this state and outputs the mean MLC positions together with a value estimate and a learned log standard deviation. At each step, the action $a_{t}$ is sampled from the distribution defined by the output MLC position means and log standard deviations. The updated dose distribution is calculated using the predicted MLC positions for the CP together with the other unchanged CP doses to get a cumulative dose distribution. The difference in dose objective value between the previous and current state is the resulting reward $r_{t}$ of that step. After the network has completed a full episode (predicted all N CPs), the collected trajectories are used to calculate advantages. MLC network weights are updated using an Adam optimizer to minimize the negative of the PPO loss function in Equation 4.

After an MLC training loop is completed, the MU network training begins. The MU network operates similarly to a segment weight optimizer, taking in all the predicted MLC positions and iterating the MU values of all CPs to find the optimal dose distribution. Like the MLC network, the MU network takes a state and predicts the MU means together with a learned log standard deviation and a value estimate. At each step, an action is sampled from the distribution defined by the output MU means and log standard deviations. The new predicted MUs are used to calculate a new cumulative dose distribution. Our IMRT dose calculator was adapted to VMAT deliveries by using a weighted sum of open field dose calculations at angles where the open field doses overlap based on the distance to each field edge. To validate this adaptation, a gamma analysis with a 3%/2 mm gamma criterion, and 10% threshold was used to compare the VMAT radiation plan dose calculated using this algorithm against the GPUMCD calculated reference plans of the 20 patient testing cohort. The resulting reward is therefore the difference in dose objective value between the previous and current state. This process is repeated 8 times per episode. The trajectories collected from this loop are used to calculate advantages and returns which are used to update the MU network weights using an Adam optimizer to minimize the negative of the PPO loss function in Equation 4. After both training loops have been completed, the predicted set of MU and MLC parameters are chosen as the new initial state if the resulting plan achieved a better reward than the previous initial state.

The overall training process involves a nested loop structure. A full training run consists of 2000 iterations and within each iteration, the algorithm processes every patient in the training cohort. For a single patient, the plan generation process is defined as an episode. An episode begins with the MLC network sequentially predicting MLC positions for all CPs to complete a full arc. Following this, the MU network takes the full set of predicted MLC positions and performs 8 inference steps to adjust the MUs for all CPs collectively. The final plan from this episode is compared to the plan used at the start of the episode; if the new plan yields a better total reward value, it becomes the new initial state for that patient in the subsequent training iteration. This ensures a progressively improving baseline for each patient throughout training.

### 2.4 Plan evaluation

Timing statistics were reported for all parts of the workflow. After training, the RL policy networks were used to create VMAT plans for a set of 20 test patients. During evaluation, the network loop was repeated as many times as needed until the plan was no longer improved, or the final plan reward no longer increased. The RL VMAT plans of the 20 test patients were compared to the reference VMAT plans created in the Monaco TPS using traditional inverse optimization methods. The evaluation compared the $D_{mean}$, $D_{max}$, and other clinically relevant dose objectives for each structure as shown in Table 2. Conformity was calculated as the ratio between the 95% prescription volume and target volume. The mean dosimetric values were compared across the reference and RL cohorts with the statistical significance of their differences, computed using Wilcoxon ranked sum tests.

**TABLE 2:** Mean ± SD dose metrics for RL generated and reference plans for the test patients. Statistically significant differences (p < 0.05) are denoted in bold with an asterisk.

Structure	Metric	Reference	RL	*p*‐value
**PTV**	$D_{mean}$ (Gy)	41.641±1.225	42.014±1.224	0.304
	$D_{2\%}$ (Gy)	47.351±2.610	48.095±2.504	*0.048
	$D_{98\%}$ (Gy)	34.935±0.600	34.587±0.669	0.224
	*CI*	1.396±0.106	1.424±0.073	0.251
**Bladder**	$D_{mean}$ (Gy)	11.414±3.988	10.578±3.754	0.417
	$V_{50\%}$ (Gy)	8.288±3.793	7.709±3.451	0.607
**Rectum**	$D_{mean}$ (Gy)	14.035±2.134	12.960±1.847	*0.020
	$V_{50\%}$ (Gy)	10.411±3.138	8.793±2.744	0.130
	$V_{80\%}$ (Gy)	3.183±1.534	2.829±1.319	0.417
**Left femoral head**	$D_{mean}$ (Gy)	8.919±1.652	8.719±1.867	0.626
	$V_{40\%}$ (Gy)	2.853±2.484	3.393±3.640	0.892
**Right femoral head**	$D_{mean}$ (Gy)	8.571±1.744	9.874±2.242	*0.045
	$V_{40\%}$ (Gy)	1.965±2.615	5.593±5.106	*0.020

## 3 RESULTS

### 3.1 Workflow timing

Before training began, network inputs and patient‐specific open‐fields^30^ needed to be calculated from dicom files exported from the TPS. In Monaco, these open fields took 24.2±6.0 s to calculate at 5 mm isotropic grids. After exporting the open‐field doses in addition to patient structure files, input generation took an additional 22.4±8.3 s per patient on average. After these inputs were generated, the RL plans were generated by the MLC and MU networks in 6.3±4.7 s on a single Telsa V100 GPU. The large variance in plan generation time was due to the varying amount iterations run during inference. Each patient began another iteration through each network as long as their cumulative reward continued to increase. Some patients required only two passes through the network, while others saw continual improvement for up to 8 iterations.

### 3.2 Training

**FIGURE 3:**
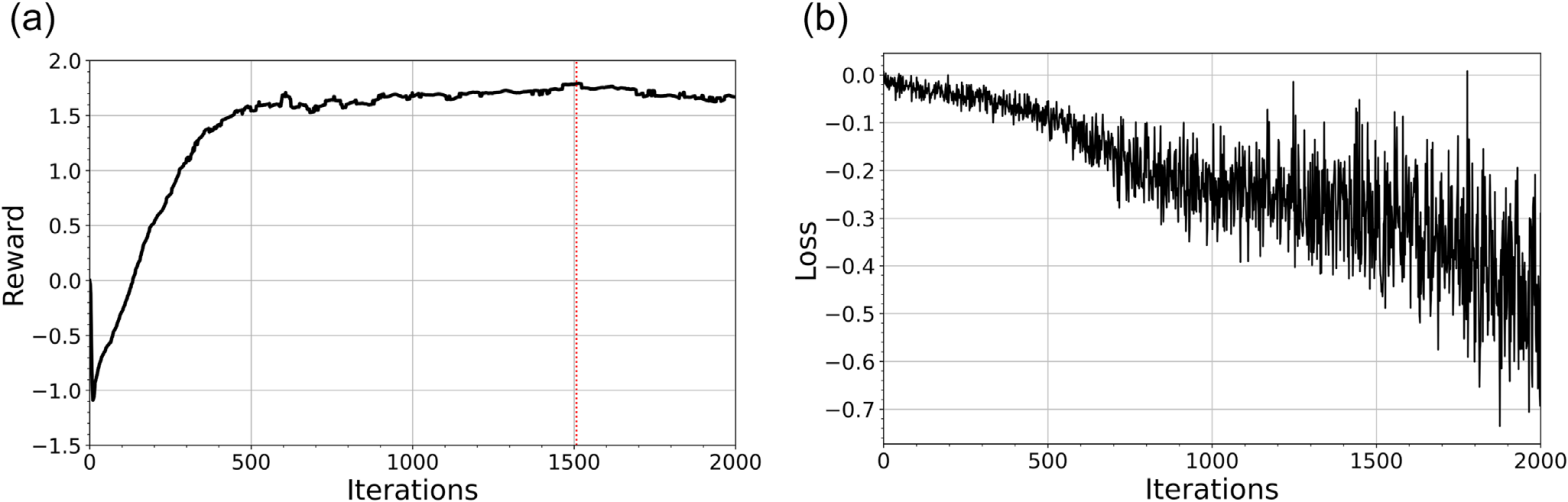
(a) Plot of mean total plan reward versus RL iteration number for patients in the validation cohort during training. The dotted red line shows the iteration where the mean total plan reward was the highest. (b) Plot of the negative total policy loss versus RL training iteration for the MLC network.

The IL process used to initialize network weights took 32 min for the 500 epochs of supervised learning‐based training. Training of the RL network for 2000 iterations took 71 h to complete on our 8 GPU cluster. The relevant training metrics are shown in Figure 3. On average, the total plan reward increased steadily, reaching a maximum at iteration 1506, shown in Figure 3a. Network weights from this iteration were used during inference on the patient test set.

Figure 3b shows negative loss versus RL training iteration. As stated in section 2.2.3, this behavior is expected in PPO training where we are aiming to minimize this quantity over the course of training.

### 3.3 Plan evaluation

**FIGURE 4:**
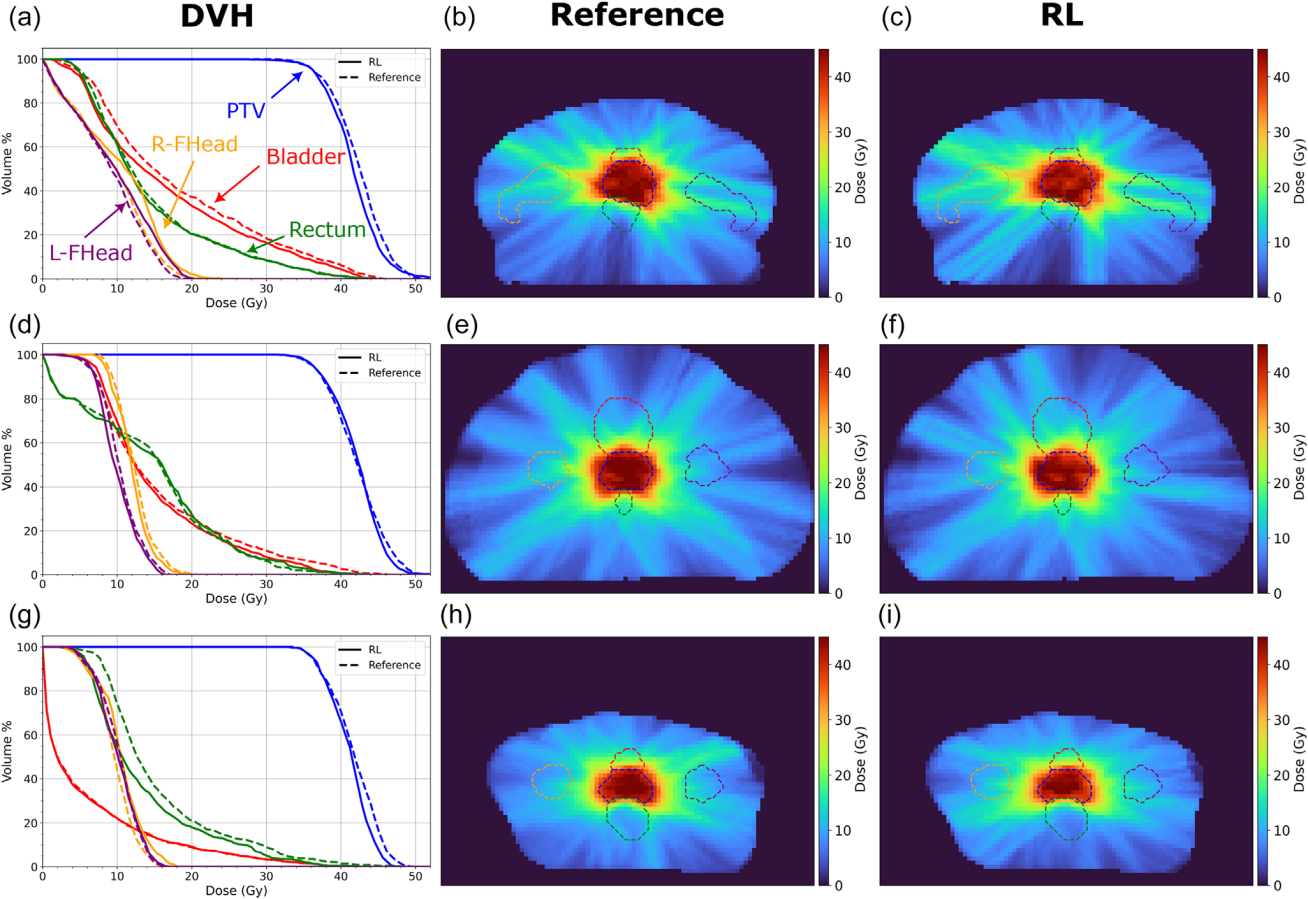
DVH of three sample patients (a, d, g) with corresponding axial slices of the reference (b, e, h) and RL generated (c, f, i) dose distributions. The target, bladder, rectum, right femoral head, and left femoral head contours are shown in blue, red, green, orange, and purple dashed lines respectively in the 2D dose views.

The evaluation of our VMAT dose calculator showed an accuracy of 95.13 ± 2.82% gamma pass rate against the Monaco GPUMCD Monte Carlo dose calculation for the testing cohort. The RL generated plans were capable of appropriately sparing the rectum and bladder while achieving the needed PTV coverage. This validates the ability of the networks to respond to the dosimetric reward and set clinically priorities. This is demonstrated in the sample cases shown in Figure 4 that include both reference and RL plan dose distributions with their corresponding DVHs. In these cases, the RL generated plans achieved similar or improved OAR sparing with similar or slightly hotter PTVs.

**FIGURE 5:**
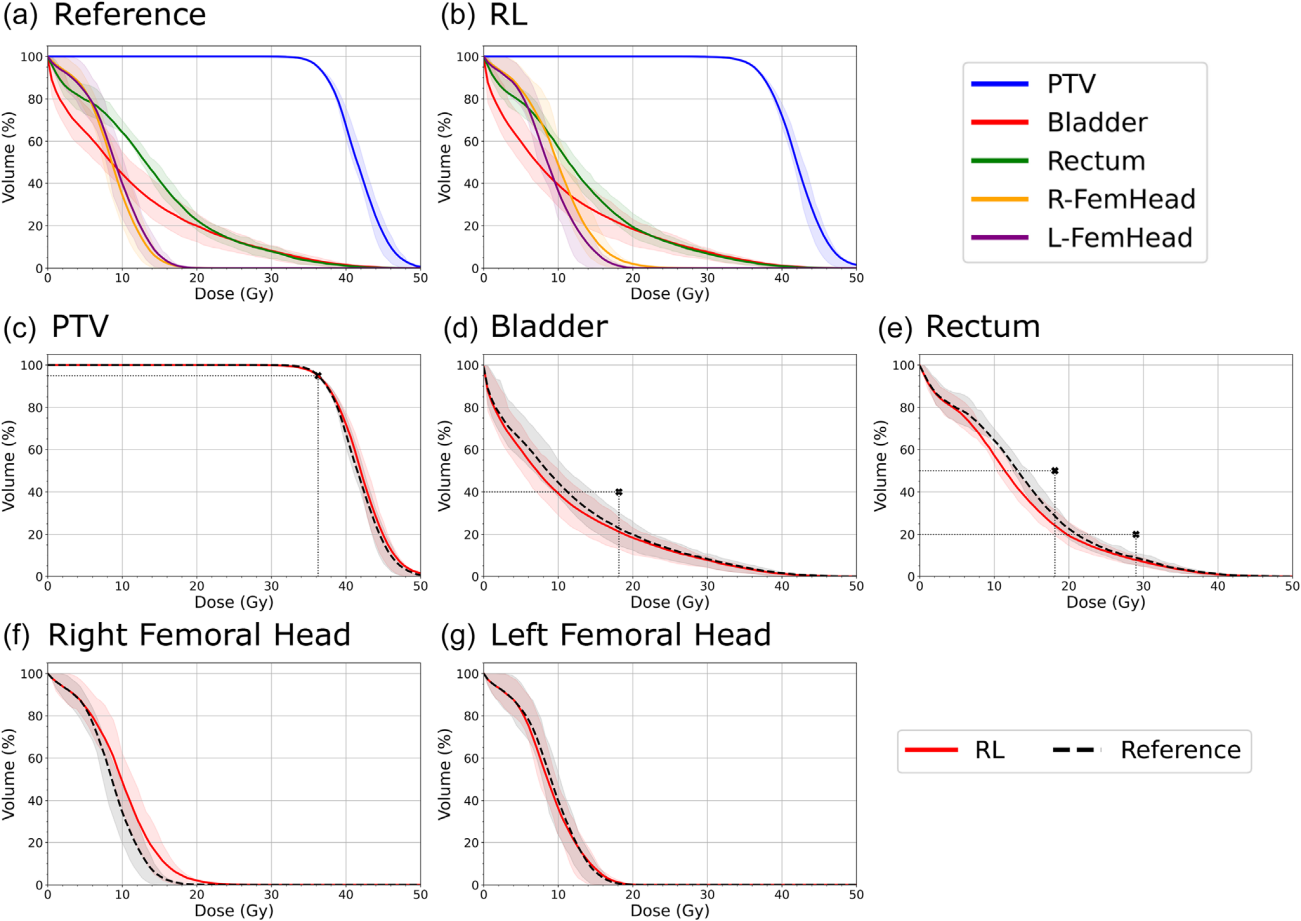
Mean DVH plots of the reference (a) and RL generated (b) plans. OAR‐specific mean DVH curves for the PTV (c), bladder (d), rectum (e), right femoral head (f), and left femoral head (g) with small black markers denoting the constraints used in the dose objective reward function. Shaded regions represent the banded interquartile range.

This trend is further observed in the DVH curves generated from the 20 patient test cohort shown in Figure 5 with the aggregate dose metrics shown in Table 2. Most of the metrics measured showed no statistically significant difference between the reference and RL plans. Of the metrics used in building the reward, only the difference in $D_{2\%}$ for the PTV and the rectum $D_{\mathrm{mean}}$ showed statistical significance, with the RL plans resulting in a slightly higher $D_{2\%}$ and slightly lower rectum $D_{\mathrm{mean}}$. Of the two femoral heads, only the right showed a statistical difference in $D_{\mathrm{mean}}$ and $V_{40\%}$, where the RL plans were hotter.

All RL generated and reference plans evaluated met or exceeded every constraint in Table 1. This further demonstrates the ability of the network to respond to the chosen dosimetric reward.

## 4 DISCUSSION

In this study, we developed a deep RL framework capable of end‐to‐end VMAT machine parameter optimization, generating clinically acceptable SBRT treatment plans for prostate cancer without a commercial TPS optimizer. Once trained the two RL networks work in tandem to generate plans in an average of approximately 6 s. The conventional VMAT planning process can often take hours and involve iterative, manual adjustments by a planner so this rapid generation time highlights the potential of our approach for online adaptive radiotherapy workflows where treatment plans must be created or modified quickly in response to daily anatomical changes. The current network uses a single arc which is sufficient for localized prostate cancer but generalizing the network to intermediate and high‐risk groups will require multiple arcs which is the subject of future work. Additionally, avoidance angles and partial arcs can easily be incorporated into our framework through the addition of a geometric constraint in the reward function. The geometric reward would penalize angles that deliver entrance dose though a prosthesis or organ to be avoided. Our tandem RL framework provides the ability to incorporate geometric rewards in addition to the dosimetric rewards presented here and is the subject of future work to generalize our framework.

The RL algorithm was able to meet all predefined clinical constraints for the PTV, bladder, and rectum. This success can likely be attributed to the design of the reward function, which was structured to heavily prioritize achieving the specific dosimetric objectives. By rewarding the agent for meeting these goals, the training process effectively taught the network to generate plans that adhere to clinical standards. This further suggests that manipulating these constraints in the reward function can encourage the network to prioritize other such constraints without needing completed treatment plans optimized to those objectives. The RL generated plans also demonstrated improved OAR sparing for both the bladder and rectum when compared to the reference plans, though this often came with slightly higher maximum doses within the PTV and right femoral head. This trade‐off suggests that the weights assigned to the OAR constraints within the reward function may have been overprioritized compared to PTV homogeneity and may benefit from additional tuning. The test plans met the mechanical constraints of the machine as well. The Unity MR‐Linac has a dose rate of 425 MU/min with a conservative MLC leaf speed of 6 cm/s and all predicted RL plans predicted MLC leaf positions and MU values that meet these constraints. While the current framework predicts MUs delivered per CP at set gantry angles, future iterations could predict dose rate at variable angles subject to the minimum and maximum constraints of the treatment machine. The MLC and MU information can be formatted into a DICOM RT plan file for importing into a TPS, but currently the clinical Monaco TPS does not allow for external DICOM RT plan files to be imported and recalculated for the Unity MR‐Linac beam model. However, once this function is available, and VMAT becomes clinically available on the Unity MR‐Linac system, we plan on performing these tests as further validations as the subject of future work.

The use of a tandem‐network architecture was a deliberate design choice to effectively manage the complexity of the VMAT optimization task. The MLC network utilized beam's‐eye‐view inputs, which provided the necessary spatial context to find the optimal aperture shape for each individual CP. In contrast, the MU network was designed to take a global perspective, assessing the cumulative dose distribution and MLC positions from all CPs to modulate the intensity of segment. This separation of duties allowed the MLC network to focus on spatial modulation and the MU network to determine the optimal doses for the given MLC positions. This comes with the downside of requiring significantly more computational resources to train two separate networks simultaneously.

This study also has a few limitations. First, the reward function was limited to constraints for the PTV, bladder, and rectum to simplify the initial training process, as sequentially training two networks is inherently complex. This framework could be expanded to include additional OARs or more nuanced PTV objectives, though this would necessitate careful retuning of the reward function to ensure stable and effective training. There are also notable limitations that relate to the dose calculator^30^ used to determine the dosimetric rewards and input states. Both the input states and rewards are critically important parts of the training process, meaning that the performance of the RL network is directly linked to the performance of the dose calculation engine. Currently, the doses are predicted on 5 mm grids due to GPU memory limitations for training, but smaller 2–3 mm grids could be achieved with GPU processors with more memory (e.g., Tesla NVIDIA RTX Pro 6000 Blackwell Max‐Q GPUs with up to 96 GB memory). A finer resolution dose calculation would likely result in higher quality features from each of the networks as well as a more accurate reward signal for training. Additionally, current algorithm relies on Monte Carlo open field dose calculations which adds significant overhead time to the treatment plan prediction. There may be ways to improve this time, but this is the subject of future work. Pre‐processing the data for inputs into our networks can also be significantly improved by shifting the pre‐processing work from the CPU to the GPU. The current work also does not include dynamic jaw tracking which would act to further trim the edges of the MLC field, which is also the subject of future work.

## 5 CONCLUSION

We developed and validated a novel deep RL framework for the end‐to‐end optimization of VMAT treatment plans. Our approach demonstrates that an RL agent can rapidly generate clinically acceptable plans for prostate cancer that are dosimetrically comparable to those produced by conventional methods without the need for a commercial optimizer. The RL approach allows for significant generalizability and doesn't rely on the quality of previously generated treatment plans. Our proposed auto planning process generates a treatment plan in an average of less than 30 s (22.4±8.3 s for input pre‐processing and 6.3±4.7 treatment plan prediction) that meets the physical constraints of the machine. The speed and adaptability offered by this method make it a promising tool for auto‐planning, which is of growing importance in adaptive radiotherapy workflows where both speed and consistency are crucial. In the future we aim to incorporate patient‐specific clinical goals, further advancing the potential of RL to create more personalized treatment planning solutions.

## CONFLICT OF INTEREST STATEMENT

Joel St‐Aubin acknowledges a research agreement with Elekta AB, Stockholm not directly pertaining to this work. All other authors have no pertinent conflicts of interest to report.

## References

[1] Palma D , Vollans E , James K , et al. Volumetric modulated arc therapy for delivery of prostate radiotherapy: comparison with intensity‐modulated radiotherapy and three‐dimensional conformal radiotherapy. Int J Radiat Oncol Biol Phys. 2008;72(4):996‐1001. doi:10.1016/j.ijrobp.2008.02.047 18455326

[2] Unkelbach J , Bortfeld T , Craft D , et al. Optimization approaches to volumetric modulated arc therapy planning. Med Phys. 2015;42(3):1367‐1377. doi:10.1118/1.4908224 25735291 PMC5148175

[3] Men C , Romeijn HE , Jia X , Jiang SB . Ultrafast treatment plan optimization for volumetric modulated arc therapy (VMAT). Med Phys. 2010;37(11):5787‐5791. doi:10.1118/1.3491675 21158290

[4] Tian Z , Peng F , Folkerts M , Tan J , Jia X , Jiang SB . Multi‐GPU implementation of a VMAT treatment plan optimization algorithm. Med Phys. 2015;42(6):2841‐2852. doi:10.1118/1.4919742 26127037

[5] Peng F , Jia X , Gu X , Epelman MA , Romeijn HE , Jiang SB . A new column‐generation‐based algorithm for VMAT treatment plan optimization. Phys Med Biol. 2012;57(14):4569. doi:10.1088/0031-9155/57/14/4569 22722760

[6] Güngör G , Serbez İ , Temur B , et al. Time analysis of online adaptive magnetic resonance‐guided radiation therapy workflow according to anatomical sites. Pract Radiat Oncol. 2021;11(1):e11‐e21. doi:10.1016/j.prro.2020.07.003 32739438

[7] Tengler B , Künzel LA , Hagmüller M , et al. Full daily re‐optimization improves plan quality during online adaptive radiotherapy. Phys Imaging Radiat Oncol. 2024;29:100534. doi:10.1016/j.phro.2024.100534 38298884 PMC10827578

[8] Wang C , Zhu X , Hong JC , Zheng D . Artificial intelligence in radiotherapy treatment planning: present and future. Technol Cancer Res Treat. 2019;18:1533033819873922. doi:10.1177/1533033819873922 31495281 PMC6732844

[9] Shiraishi S , Moore KL . Knowledge‐based prediction of three‐dimensional dose distributions for external beam radiotherapy. Med Phys. 2016;43(1):378‐387. doi:10.1118/1.4938583 26745931

[10] Nguyen D , Jia X , Sher D , et al. 3D radiotherapy dose prediction on head and neck cancer patients with a hierarchically densely connected U‐net deep learning architecture. Phys Med Biol. 2019;64(6):065020. doi:10.1088/1361-6560/ab039b 30703760

[11] Barragán‐Montero AM , Nguyen D , Lu W , et al. Three‐dimensional dose prediction for lung IMRT patients with deep neural networks: robust learning from heterogeneous beam configurations. Med Phys. 2019;46(8):3679‐3691. doi:10.1002/mp.13597 31102554

[12] Chen X , Men K , Li Y , Yi J , Dai J . A feasibility study on an automated method to generate patient‐specific dose distributions for radiotherapy using deep learning. Med Phys. 2019;46(1):56‐64. doi:10.1002/mp.13262 30367492 PMC7379709

[13] Boutilier JJ , Lee T , Craig T , Sharpe MB , Chan TCY . Models for predicting objective function weights in prostate cancer IMRT. Med Phys. 2015;42(4):1586‐1595. doi:10.1118/1.4914140 25832049

[14] Campbell WG , Miften M , Olsen L , et al. Neural network dose models for knowledge‐based planning in pancreatic SBRT. Med Phys. 2017;44(12):6148‐6158. doi:10.1002/mp.12621 28994459 PMC5734636

[15] Qilin Z , Peng B , Ang Q , et al. The feasibility study on the generalization of deep learning dose prediction model for volumetric modulated arc therapy of cervical cancer. J Appl Clin Med Phys. 2022;23(6):e13583. doi:10.1002/acm2.13583 35262273 PMC9195039

[16] Sutton R , Barto A . Reinforcement Learning: An Introduction. 2nd ed. A Bradford Book; 2018

[17] Mnih V , Kavukcuoglu K , Silver D , et al. Human‐level control through deep reinforcement learning. Nature. 2015;518(7540):529‐533. doi:10.1038/nature14236 25719670

[18] Vinyals O , Babuschkin I , Czarnecki WM , et al. Grandmaster level in StarCraft II using multi‐agent reinforcement learning. Nature. 2019;575(7782):350‐354. doi:10.1038/s41586-019-1724-z 31666705

[19] Silver D , Schrittwieser J , Simonyan K , et al. Mastering the game of Go without human knowledge. Nature. 2017;550(7676):354‐359. doi:10.1038/nature24270 29052630

[20] Li C , Guo Y , Lin X , Feng X , Xu D , Yang R . Deep reinforcement learning in radiation therapy planning optimization: a comprehensive review. Physica Med. 2024;125:104498. doi:10.1016/j.ejmp.2024.104498 39163802

[21] Shen C , Nguyen D , Chen L , et al. Operating a treatment planning system using a deep‐reinforcement learning‐based virtual treatment planner for prostate cancer intensity‐modulated radiation therapy treatment planning. Med Phys. 2020;47(6):2329‐2336. doi:10.1002/mp.14114 32141086 PMC7903320

[22] Abrar MM , Sapkota P , Sprouts D , Jia X , Chi Y . Actor critic with experience replay‐based automatic treatment planning for prostate cancer intensity modulated radiotherapy. Med Phys. 2025;52(7):e17915. doi:10.1002/mp.17915 40450383 PMC12258009

[23] Shen C , Chen L , Jia X . A hierarchical deep reinforcement learning framework for intelligent automatic treatment planning of prostate cancer intensity modulated radiation therapy. Phys Med Biol. 2021;66(13):134002. doi:10.1088/1361-6560/ac09a2 PMC8406431 34107460

[24] Shen C , Gonzalez Y , Klages P , et al. Intelligent inverse treatment planning via deep reinforcement learning, a proof‐of‐principle study in high dose‐rate brachytherapy for cervical cancer. Phys Med Biol. 2019;64(11):115013. doi:10.1088/1361-6560/ab18bf 30978709 PMC7014824

[25] Gao Y , Kyun Park Y , Jia X . Human‐like intelligent automatic treatment planning of head and neck cancer radiation therapy. Phys Med Biol. 2024;69(11):115049. doi:10.1088/1361-6560/ad4b90 PMC11148880 38744304

[26] Pu G , Jiang S , Yang Z , Hu Y , Liu Z . Deep reinforcement learning for treatment planning in high‐dose‐rate cervical brachytherapy. Phys Med. 2022;94:1‐7. doi:10.1016/j.ejmp.2021.12.009 34959169

[27] Hrinivich WT , Bhattacharya M , Mekki L , et al. Clinical VMAT machine parameter optimization for localized prostate cancer using deep reinforcement learning. Med Phys. 2024;51(6):3972‐3984. doi:10.1002/mp.17100 38669457

[28] Kontaxis C , Woodhead PL , Bol GH , Lagendijk JJW , Raaymakers BW . Proof‐of‐concept delivery of intensity modulated arc therapy on the Elekta Unity 1.5 T MR‐linac. Phys Med Biol. 2021;66(4):04LT01. doi:10.1088/1361-6560/abd66d 33361560

[29] van As N , Griffin C , Tree A , et al. Phase 3 trial of stereotactic body radiotherapy in localized prostate cancer. N Engl J Med. 2024;391(15):1413‐1425. doi:10.1056/NEJMoa2403365 39413377 PMC7616714

[30] Shaffer N , Snyder J , St‐Aubin J . Validation of a rapid algorithm for repeated intensity modulated radiation therapy dose calculations. Biomed Phys Eng Express. 2024;11(1):015046. doi:10.1088/2057-1976/ad9f6a 39681005

[31] Pignatelli E , Ferret J , Geist M , et al. a survey of temporal credit assignment in deep reinforcement learning. arXiv. 2024. doi:10.48550/arXiv.2312.01072. Preprint posted online.

[32] Minsky M . Steps toward Artificial Intelligence. Proc IRE. 1961;49(1):8‐30. doi:10.1109/JRPROC.1961.287775

[33] Schulman J , Moritz P , Levine S , Jordan M , Abbeel P . High‐dimensional continuous control using generalized advantage estimation. *arXiv*. 2018. doi:10.48550/arXiv.1506.02438. Preprint posted online October 20 2018.

[34] Schulman J , Wolski F , Dhariwal P , Radford A , Klimov O . Proximal policy optimization algorithms. arXiv. 2017. doi:10.48550/arXiv.1707.06347. Preprint posted online August 28 2017.

[35] Mnih V , Badia AP , Mirza M , et al. Asynchronous methods for deep reinforcement learning. arXiv. 2016. doi:10.48550/arXiv.1602.01783. Preprint posted online June 16 2016.

[36] Williams RJ . Simple statistical gradient‐following algorithms for connectionist reinforcement learning. Mach Learn. 1992;8(3):229‐256. doi:10.1007/BF00992696

[37] Terven J . Deep reinforcement learning: a chronological overview and methods. AI. 2025;6(3):46. doi:10.3390/ai6030046

[38] Hussein A , Gaber MM , Elyan E , Jayne C . Imitation learning: a survey of learning methods. ACM Comput Surv. 2017;50(2):1‐35. doi:10.1145/3054912

[39] Florence P , Lynch C , Zeng A , et al . Implicit Behavioral Cloning. In: Proceedings of the 5th Conference on Robot Learning. PMLR; 2022:158‐168. Accessed September 8, 2025. https://proceedings.mlr.press/v164/florence22a.html

[40] Choi T , Cho K , Sung Y . Approaches that use domain‐specific expertise: behavioral‐cloning‐based advantage actor‐critic in basketball games. Mathematics. 2023;11(5):1110. doi:10.3390/math11051110

